# The phenomenon of Type A and B personality prevalence and their correlation to the anti-health behavior of Polish physicians

**DOI:** 10.3389/fpsyg.2025.1608564

**Published:** 2025-08-12

**Authors:** Józefa Dąbek, Sławomir Wojczyk, Julia Bijoch, Oskar Sierka

**Affiliations:** ^1^Department of Cardiology, Faculty of Health Sciences in Katowice, Medical University of Silesia, Katowice, Poland; ^2^Department of Vascular and General Surgery, Municipal Specialist Hospital No. 4 in Bytom, Bytom, Poland; ^3^Collegium Medicum, Faculty of Medicine, WSB University, Dąbrowa Górnicza, Poland; ^4^College of Doctoral School, Faculty of Medical Sciences in Katowice, Medical University of Silesia, Katowice, Poland

**Keywords:** personality type, physicians, risk factors, unhealthy behaviors, relationship

## Abstract

**Objectives:**

Personality is connected with the possible development of various diseases; therefore, the aim of this study was to assess the occurrence of Type A and B personalities and their relationship with anti-health behaviors among Polish doctors.

**Methods:**

The study group included 823 (100%) physicians working in Poland. The study was conducted using an original questionnaire, consisting of questions regarding general data, and standardized anti-health behaviors assessment questionnaires available publicly.

**Results:**

The most common risk factors in the study group were: stress (545; 66.22%), insufficient sleep (375; 45.57%) and low physical activity (322; 39.17%). Physicians with Type A personality achieved higher score values on all subscales of the My Eating Habits Questionnaire than physicians with intermediate and Type B personalities.

**Conclusion:**

The occurrence of Type A personality was associated with, among others, worse eating habits, more frequent sleep deprivation, and exposure to stress and low physical activity. There is a need to conduct educational activities at an early school stage regarding the benefits of following healthy lifestyle recommendations, and consequently limiting the development of diseases and their complications.

## 1 Background

According to the American Psychological Association, the meaning of personality refers to the preserved characteristics and behavior that characterize a person’s unique adjustment to life, including major traits, interests, drives, values, self-concept, abilities, and emotional patterns (American Psychological Association, 2024). Personality plays a key role in human behavior and influences academic performance, social standing, career stability, and relationships with peers and family. It is one of the main determinants of a person’s lifestyle. It also refers to the specific combination of talents, values, hopes, loves, hates, and habits that make each person unique (Bhattacharjee and Ramkumar, 2025).

In the course of the research, personalities that have a positive and negative impact on human health were distinguished. The characteristics of people with Type A personality are: openness, a high level of ambition, good organization of one’s work, impatience and proactivity, as well as choleric and feeling constant anxiety that forces one to take action. People with the described personality are often workaholics and constantly strive for perfection and hate delays and ambivalence. Unfortunately, the above features harm their experience of stress and lead to quick boredom with work and the possibility of developing adverse health complications if the occurrence of additional risk factors for circulatory system diseases is taken into account (Samaras and Galanakis, 2022).

Moreover, mentioned personality can lead to burnout syndrome. Mentioned condition is characterized by extreme fatigue, reduced ability to regulate cognitive and emotional processes, and psychological distance, which, according to various statistics, affects over 10% of employees in various professions and sectors (Hendrikx et al., 2024).

Eating behavior can be defined as the result of conscious, collective and repetitive behavior leading individuals to select, prepare, consume and use specific food products or diets in response to social, cultural and religious influences (Fisberg et al., 2024). Eating disorders have become a growing social, research and clinical problem in recent years. The number of cases of anorexia nervosa and bulimia, as well as the opposite side, overweight and obesity, is increasing (Feng et al., 2023). Overweight and obesity, classified as lifestyle diseases, contribute to the development of cardiovascular diseases and cancers, which are among the leading causes of death and disability worldwide, affecting the populations of both developed and developing countries (Cena and Calder, 2020). Although genetic factors are known to contribute to the development of these diseases, most of them are caused by the occurrence of unfavorable modifiable factors related mainly to lifestyle, including an improper diet. They play a large role in the development and progression of circulatory system diseases, developing based on atherosclerosis (Kolb and Martin, 2017). Studies have shown that improper dietary choices contribute to the risk of developing diseases such as coronary heart disease, hypertension and hypercholesterolemia, and the observed increase in the incidence of chronic lifestyle diseases is causally related to global dietary patterns, which are becoming more and more “Western” - high-energy, processed food, animal origin, alcoholic beverages, and to a lesser extent, vegetables, fruits and nuts (Clemente-Suárez et al., 2023). The quantity and quality of food consumed affect human health. While the quality of selected meals varies from person to person and depends on various factors, including cultural/religious conditions, biological clocks play an important role in the consumption of the quantity of meals, coordinating the time of many daily behaviors (e.g., sleep/wakefulness, eating/fasting) and physiology (e.g., hormone release, heart function) (Manoogian et al., 2019). These mechanisms take into account signals from the external environment to coordinate internal biology with the environment. The presence of interfering signals, e.g., stress or exposure to light during sleep, adversely affects the processes taking place in the body, leading to their deregulation.

It should also be emphasized that some circadian rhythms are regulated by eating meals, and eating at fixed times becomes the foundation for their proper functioning. Mentioned rhythms play a crucial role in a wide range of physiological and behavioral processes. The human body’s master “clock,” located in the suprachiasmatic nucleus of the hypothalamus, regulates all other circadian rhythms by synchronizing numerous environmental signals, e.g., light and temperature (Lan et al., 2024). Consequently, drastic changes in meal times from day to day weaken their physiological effects, negatively affecting a person’s health.

Other factors influencing the development of cardiovascular diseases are, among others: alcohol consumption, tobacco smoking, low physical activity, sleeping disorders and exposure to severe stress.

Mentioned factors and illness-prone personality types are often present among physicians who work in several places, for a long time, and struggle with difficulties while helping others. In addition, a constant lack of time and a large number of duties, as well as stress, contribute to choosing easy and quick ways of “pressure release,” e.g., smoking/drinking alcohol, which are harmful to health. This study takes into account anti-health behaviors (risk factors of various diseases) because identification of their presence is crucial for prediction, prevention, and effective intervention of various health outcomes and behavioral problems. Moreover, understanding risk factors origins, presence, and occurrence is crucial in making tailored interventions and mitigating negative outcomes among various society groups.

## 2 Objectives

The study aimed to assess the occurrence of Type A and B personalities and their relationship with anti-health behaviors of Polish physicians.

## 3 Materials and methods

The study was initiated after the research project was accepted by the Bioethics Committee of the Medical University of Silesia in Katowice based on the decision PCN/0022/KB/287/19 of 16 December 2019, taking into account the applicable regulations and requirements for conducting scientific research.

The whole study group included 876 physicians working in Poland. The inclusion criteria for the study were: expressing informed and voluntary consent to participate in the study, the ability to complete the survey questionnaire, and working in the profession for at least 1 year. Based on correctness of provided responses a subgroup of 823 (100%) physicians was selected, whose responses were analyzed in the article below.

The study was conducted using an original questionnaire consisting of questions regarding general data such as: age, gender, place of residence and questions regarding the characteristics of the physicians’ professional work and their behaviors. In this study assessment of anti-health behaviors occurrence was based on self-reported responses provided by physicians as well as standardized questionnaires.

In order to assess personality type, the *Framingham Type A Scale* questionnaire by Chesney and Rosenman, in the Polish adaptation by Z. Juczyński, was used. The aforementioned questionnaire assesses the intensity of 2 characteristics of the Type A behavior pattern: Haste and Competition. The Type A Scale consists of 10 statements, of which the first five concern characteristics and properties typical of an individual. The next four assess the feelings accompanying the respondent at the end of a typical day. The last question concerns the feeling of time pressure. The intensity of the examined features increases with the increase in point values. Based on the obtained point results, the types of behavior of the respondents can be classified – Type A or B or in intermediate.

In addition, the study used standardized questionnaires to examine the occurrence of anti-health behaviors that have a proven contribution to the development and progression of cardiovascular diseases. Each of them is briefly described below.

The correctness of the diet was assessed using *The Point Scale of the Diet Assessment Questionnaire* according to Z. Bielińska. The aforementioned scale assesses the diet understood as the frequency and regularity of eating meals and the frequency of using food products that are a source of specific nutrients. The assessment is made on the basis of awarded points; the higher the score, the better the diet. Collecting 31 or more points indicates a correct diet, while obtaining a lower score is an indication for its improvement.

*The My Eating Habits Questionnaire* (N. Ogińska-Bulik, L. Putyński) was used to measure eating habits (customs) in adults and older adolescents. Its results can be used to predict the tendency to abnormal weight gain. It consists of a total of 30 statements, to which the respondents provide answers, divided into 3 subscales: *Habitual overeating, Emotional overeating, Dietary restrictions*. A high score obtained in the above-mentioned subscales and the total number of points obtained indicates improper eating habits, i.e., a tendency to overeat or refrain from eating. The described questionnaire also allows for the assessment of the degree of risk of overweight or obesity in people who maintain a healthy body weight thanks to dietary restrictions, and represent eating habits and a psychological structure characteristic of people with obesity.

Participants were recruited from the researchers’ colleagues and acquaintances. In addition, they received additional questionnaires for further distribution to other physicians (snowball sampling method). Questionnaires were distributed only in paper form, and all participants were self-administering them. Each study participant received the questionnaire in a white, unmarked envelope. After completing the survey, the physicians placed the questionnaire and sealed them in the attached envelope, preventing their identification. The envelopes were collected and placed in an unmarked box, to which only the researchers had access. The box with the answers was opened only when the information from the collected questionnaires was entered into the database prepared for the study. During the study, 900 questionnaires were distributed, of which 876 (97.33%) were returned to the research team. Of these, 823 (93.95%) were included in the analyses as correctly completed.

Statistical analyses were performed using Statistica 13.3 software (Statsoft, Poland). Qualitative data were presented taking into account the number and percentages in relation to the entire group. Quantitative data were presented taking into account descriptive statistics, including: mean, standard deviation and median. The aforementioned data were analyzed using the Shapiro–Wilk test to assess the occurrence of a normal distribution. Due to the deviation of the described data from the normal distribution, nonparametric tests were used. The Mann–Whitney U test was used for comparisons between two groups, and the Kruskal-Wallis ANOVA test was used between more than two groups. Chi^2^ test was also used to tests of significance of differences between compared groups. Authors are aware of the multiple comparisons issue which is an increased probability of false positives of the results. Statistical significance was set at *p* < 0.05.

## 4 Results

Table 1 presents the general characteristics of the studied group of physicians.

**Table 1:** General characteristics of the studied group of doctors.

Study group of physicians (823; 100%)		
Variables	*n*	%
Sex		
Women	502	60.34
Men	321	38.58
Age [years]		
≤30	204	24.79
31–40	310	37.67
41–50	172	20.90
≥51	137	16.65
Length of service [years]		
1–10	456	54.81
11–20	176	21.15
21–30	127	15.26
>31	64	7.69
Ward type		
Surgical	273	32.81
Non-surgical	550	66.11
Additional employment		
Yes	570	68.51
No	253	31.49
Working on duty		
Yes	653	78.49
No	170	20.43

n, number of participants.

Over 60% (502; 60.34%) of the surveyed group were women, and over half of the surveyed (456; 54.81%) respondents had been working in their profession for 1 to 10 years.

The characteristics of the studied group of physicians, taking into account the occurrence of Type A/B behavior patterns and sociodemographic characteristics, were presented in Table 2.

**Table 2:** Characteristics of the studied group of doctors, taking into account the occurrence of Type A/B behavior patterns and sociodemographic characteristics.

Variables	Study group of physicians (823; 100%) personality								Chi^2^	*p*	*V*
	Type A (*n* = 349; 42.41%)		Intermediate type (*n* = 277; 33.66%)		Type B (*n* = 197; 23.94%)		SUM (*n* = 823; 100%)				
	Number (n) and percent (%) of participants										
	*n*	%	*n*	%	*n*	%	*n*	%			
Sex											
Women	226	64.76	164	59.21	112	56.85	502	61.00	3.869	0.144	0,07
Men	123	35.24	113	40.79	85	43.15	321	39.00			
Age [years]											
≤30	93	26.65	76	27.44	35	17.77	204	24.79	24.886	<0.001*	0,12
31–40	150	42.98	96	34.66	64	32.49	310	37.67			
41–50	54	15.47	58	30.69	60	30.46	172	20.90			
≥51	52	14.90	47	16.97	38	19.29	137	16.65			
Length of service [years]											
1–10	220	63.04	147	53.07	89	45.18	456	55.41	18.529	<0.01*	0,04
11–20	63	18.05	65	23.47	48	24.37	176	21.39			
21–30	46	13.18	42	15.16	39	19.80	127	15.43			
≥31	20	5.73	23	8.30	21	10.66	64	7.78			
Ward type											
Surgical	123	35.24	86	31.05	64	32.49	273	33.17	1.282	0.527	0,09
Non-surgical	226	64.76	191	68.89	133	67.51	550	66.83			
Additional employment											
Yes	259	74.21	191	68.89	120	60.91	570	69.26	10.478	<0.01*	0,11
No	90	25.79	86	31.05	77	39.09	253	30.74			
Working on duty											
Yes	292	83.67	213	76.90	148	75.13	653	79.34	7.132	0.03*	0,11
No	57	16.33	64	23.10	49	24.87	170	20.66			

n, number of participants; Chi2, value of the Chi2 statistic; *p*, level of statistical significance; V, Crammer’s *V*.

The Type A behavior pattern was demonstrated by over 40% (349; 42.41%) of the respondents and was significantly more frequent among people aged 31–40 years, as well as among respondents declaring working on duty and employed in more than one place of work.

The characteristics of the study group, taking into account the risk factors for cardiovascular diseases reported by physicians, were presented in Table 3.

**Table 3:** Characteristics of the study group, taking into account the risk factors for cardiovascular diseases reported by physicians.

Risk factors reported by physicians		Study group of physicians (823; 100%) personality										
		Type A (*n* = 349; 42.41%)		Intermediate type (*n* = 277; 33.66%)		Type B (*n* = 197; 23.94%)		SUM (*n* = 823; 100%)		Chi^2^	*p*	*V*
		*n*	%	*n*	%	*n*	%	*n*	%			
Early occurrence of circulatory system diseases in the family	Yes	51	14.61	47	16.97	34	17.26	132	16.04	0.922	0.631	0.03
	No	298	85.39	230	83.03	163	82.74	691	83.96			
Arterial hypertension	Yes	44	12.61	41	14.80	35	17.77	120	14.58	2.707	0.258	0.06
	No	305	87.39	236	85.20	162	82.23	703	85.42			
Diabetes	Yes	9	2.58	7	2.53	3	1.52	19	2.31	0.711	0.700	0.03
	No	340	97.42	270	97.47	194	98.48	804	97.69			
Tobacco smoking	Yes	52	14.90	35	12.64	17	8.63	104	12.64	4.484	0.106	0.07
	No	297	85.10	242	87.36	180	91.37	719	87.36			
Low physical activity	Yes	162	46.55	104	37.55	56	28.43	322	39.17	17.808	<0.001	0.15
	No	186	53.45	173	62.45	141	71.57	500	60.83			
Alcohol overuse	Yes	11	3.15	10	3.61	8	4.06	29	3.52	0.315	0.854	0.02
	No	338	96.85	267	96.39	189	95.94	794	96.48			
Stress	Yes	258	73.93	178	64.26	109	55.33	545	66.22	20.184	<0.001	0.16
	No	91	26.07	99	35.74	88	44.67	278	33.78			
Overweight/obesity	Yes	50	14.33	26	9.39	12	6.09	88	10.69	9.689	<0.01*	0.11
	No	299	85.67	251	90.61	185	93.91	735	89.31			
Hypercholesterolemia	Yes	45	12.89	44	15.88	22	11.17	111	13.49	2.378	0.305	0.05
	No	304	87.11	233	84.12	175	88.83	712	86.51			
Lack of sleep	Yes	194	55.59	120	43.32	61	30.96	375	45.57	31.627	<0.001	0.20
	No	155	44.41	157	56.68	136	69.04	448	54.43			

n, number of participants; Chi2, value of the Chi2 statistic; p, level of statistical significance; V, Crammer’s *V*.

The most common risk factors in the study group were: stress (545; 66.22%), insufficient sleep (375; 45.57%) and low physical activity (322; 39.17%). Taking into account the studied personality types and the occurrence of selected risk factors for cardiovascular system diseases, the Chi^2^ test showed that respondents with Type A personality more often declared lack of sleep than respondents with other personality types. Moreover, similar observations were made taking into account: the occurrence of exposure to stress. Low physical activity and being overweight/obese.

The characteristics of the study group, including the assessment of the daily menu conducted with use of *The Point Scale of the Diet Assessment Questionnaire* and descriptive statistics of the number of points obtained by the respondents, are presented in Supplementary Table S1 and Supplementary Table S2 presents the characteristics of the study group, taking into account the comparison of the number of points obtained in *The Point Scale of the Diet Assessment Questionnaire* depending on the demonstrated personality type.

Almost 46% (382; 45.91%) of the examined group of doctors declared eating 4–5 meals a day, and the presence of vegetables or fruit in 2 meals – less than 65% (539; 64.78%). Unfortunately, in all respondents (823; 100%) the applied questionnaire for assessing the diet showed a necessity of diet changes. Analyses of differences showed that, taking into account the number of points in the diet assessment questionnaire, significantly lower results, and consequently a worse diet, were obtained by physicians with Type A personality.

Supplementary Table S3 presents the characteristics of the study group, taking into account the descriptive statistics of the points obtained by the examined physicians in the individual scales of the *My Eating Habits questionnaire* and the total number of points and the characteristics of the study group of physicians, taking into account the comparison of eating habits measured with *My Eating Habits Questionnaire* and socio-demographic characteristics, are presented in Supplementary Table S4. Post-hoc analyses of the Kruskal-Wallis test for significant multiple comparisons are presented in Supplementary Tables S5, S6.

The Mann–Whitney *U* test showed that women had a greater tendency to emotional overeating (*Z* = –3.076; *p* = 0.002) and dietary restrictions (*Z* = –3.02; *p* = 0.003) than men. In addition, the aforementioned test showed that doctors working in non-surgical wards had a greater tendency to dietary restrictions (*Z* = 2.1; *p* = 0.03), and working on duty was associated with habitual overeating (*Z* = –2.28; *p* = 0.023). The Kruskal-Wallis’s test showed that people aged ≤30 years had a greater tendency to habitual overeating than respondents aged 41–50 (*p* < 0.001) and ≥51 years (*p* = 0.002), and also revealed that people who had been working for 1 to 10 years had a greater tendency to habitual overeating than the remaining respondents (11–20 – *p* < 0.001, 21–30 – *p* = 0.003, ≥31 – *p* = 0.04).

The characteristics of the study group, taking into account the number of points obtained in the *My Eating Habits Questionnaire* and the occurrence of Type A/B personality, were presented in Table 4.

**Table 4:** Characteristics of the study group, taking into account the number of points obtained in the *My Eating Habits Questionnaire* and the occurrence of Type A/B personality.

Variables	Study group of physicians (823; 100%) personality														
	Type A (*n* = 349; 42.41%)					Intermediate type (*n* = 277; 33.66%)					Type B (*n* = 197; 23.94%)				
	*M*	SD	Me	Q1	Q3	M	SD	Me	Q1	Q3	*M*	SD	Me	Q1	Q3
*Habitual overeating*	3.26	2.58	3.00	1.00	5.0	2.81	2.34	2.00	1.00	4.00	2.36	2.12	2.00	1.00	4.00
*H* = 15,645; *p* < 0.001 *Post-hoc* test: A: B – *p* < 0.001															
*Emotional overeating*	4.34	2.42	4.00	2.00	6.0	3.64	2.39	3.00	2.00	5.00	3.08	2.33	2.00	1.00	5.00
*H* = 38.774; *p* < 0.001 *Post-hoc* test: A: A/B – *p* < 0.001; A: B – *p* < 0.001; A/B: B – *p* = 0.03															
*Dietary restrictions*	3.26	2.33	3.00	1.00	5.0	2.78	2.30	2.00	1.00	4.00	2.79	2.18	2.00	1.00	4.00
*H* = 9.514; p < 0.01 *Post-hoc* test: A: B – *p* < 0.01															
*Sum of points*	10.9	5.74	10.0	6.00	15.	9.22	5.71	8.00	5.00	13.00	8.23	5.35	8.00	4.00	12.0
*H* = 30.584; *p* < 0.001 Test post-hoc: A: A/B – *p* < 0.001; A: B – *p* < 0.001															

M, mean; SD, standard deviation; H, Kruskal-Wallis test value; p, level of statistical significance; A, respondents with Type A personality; A/B, respondents with intermediate personality type; B, respondents with type B personality.

Physicians with Type A personality achieved higher score values on all subscales of the *My Eating Habits Questionnaire* and on the overall score value than physicians with intermediate and Type B personalities.

## 5 Discussion

At the beginning, it has to be stated clearly that the categorization of personalities into Type A and B was criticized by various researchers. They often point out that the mentioned categorizations are an oversimplification of human personality and lack clear boundaries between categories, which can potentially lead to misclassification or stereotyping. On the other hand, the mentioned categorization is useful for understanding individuals’ work styles, team dynamics, and potential areas for personal development and stress management. This framework helps to recognize how different workers’ psychological traits might affect their performance, relationships, and well-being in the work team. Therefore, it holds value in the fact that as one of the first scales examining human personality began a discussion and research on different personality types, how they interact, and their differences.

Among study participants presence of physicians with Type A personality was not negligible. Moreover, it was proven that participants with the mentioned personality were more likely to work on duty and work in more than one place. The observed results seem to confirm the theory that people with the Type A personality are proactive and highly committed to their work. Unfortunately. many employees experience inner conflicts related to work and family. Garavan T. et al.’s study of 305 managers from 15 information and communication organizations in India found that Type A individuals are characterized by gradual, broadly defined resource depletion (family bonds, time), while Type B respondents are associated with resource gain (Garavan et al., 2021).

Eating behaviors, like personality, are shaped at different stages of life and may directly or indirectly affect health in the short and long term (Hashemi et al., 2018). What interesting, recent studies suggests that both fathers and mothers play a crucial role in shaping early children eating behaviors which are continued in adulthood (Ng et al., 2025).

Engaging in behaviors that promote proper nutrition may help reduce the incidence of lifestyle diseases, reduce the risk of premature death, and improve both physical and mental health (Sala et al., 2020). The study showed the need to improve the diet of all surveyed physicians. Moreover, it was found that those with Type A personality had worse diets than physicians from other groups. The observations obtained may be caused by the characteristics of the profession (large number of duties, lack of time to prepare properly balanced meals) or be related to the lack of knowledge in the discussed area. The study by Aggarwal M. et al. conducted in a group of over 300 doctors showed that despite the consistent opinions of the respondents regarding the importance of proper nutrition in maintaining health, only 25% correctly determined the recommended number of fruit and vegetable portions for daily consumption, and even fewer - 20%, were aware of the recommended daily limit of added sugar in meals for adults (Aggarwal et al., 2019). Research by Tapeshkin N. et al. showed that the main factors determining physicians’ choice of food products were taste preferences (50.00%) and price of the food product (33.00%). Analysis of the variation in food consumption showed that meat, vegetables, pasta, and cereal products were consumed daily by 39–56% of the surveyed physicians (Tapeshkina et al., 2024).

A conducted study showed that women had a greater tendency towards emotional overeating and dietary restriction than men. Emotional eating – the tendency to eat in response to positive and/or negative emotions – occurs in more than half of patients treated for obesity and is particularly common among women. Studies suggest that women are also more susceptible to mood and eating disorders, but the reported gender differences in how different emotions affect food intake have not been fully explored (Anversa et al., 2021). Feraco A. et al.’s study of gender differences in food preferences found that men more often preferred red and processed meat than women. Women, on the other hand, showed a greater preference for vegetables, whole grains, tofu, and dark chocolate with a high cocoa content — healthier food choices. The aforementioned study also found that women tended to eat more frequently and reported higher levels of hunger in the morning. Differences also apply to episodes of uncontrolled eating without feeling hungry, with women reporting these behaviors more often than men, especially after accounting for emotional factors (Feraco et al., 2024). Important role in emotional overeating or restrictions can be contributed to the social media influence. Research indicates strong association between improper eating behaviors and exposure of unhealthy food advertisement (Prybutok et al., 2024).

In a conducted study, it was shown that physicians aged ≤30 years had a greater tendency to habitual overeating than doctors aged 41–50 and ≥51 years, and also that people who had been working for 1–10 years had a greater tendency to habitual overeating than other respondents. Similar results were obtained in the study by Gieniusz-Wojczyk L. et al., who found that nurses aged <31 years showed a greater tendency to habitual overeating, compared to nurses older than 30 years (Gieniusz-Wojczyk et al., 2021). They also showed that nurses who were additionally employed were characterized by significantly worse eating habits compared to those without additional employment, especially in terms of emotional overeating, which was not confirmed in the study of physicians. In a conducted study, it was also shown that physicians with Type A personality were more prone to the development of obesity and its complications – higher points values, or another eating distortion from the other side – dietary restrictions.

The Chi^2^ test showed significant differences in the number of respondents from various personality groups declaring and denying the occurrence of sleep disorders. The majority of respondents reporting sleep problems had a Type A personality. The observed results can be explained by taking into account the characteristics of the personality in question. Studies conducted by Zhou U. et al. among adults regarding the relationship between Type A personality and cerebral small vessel disease showed that Type A behavior was a risk factor for the mentioned disease among older adults and that sleep quality mediated the relationship between Type A behavior and cerebral small vessel disease. Therefore, changing Type A behavior might be helpful in improving sleep quality, which may in turn reduce the prevalence of cerebral small vessel disease (Zhou et al., 2023).

It was also shown that more Type A personality physicians than in other groups were exposed to severe stress. Observed results can once again be explained by inner characteristics of the described personality, but also by characteristics of the physician’s work. They are also disturbing and indicate a real health risk. A study conducted by Miličić D. et al. aimed to define the stress level exposure in the 5 years before acute myocardial infarction (AMI) in correlation with Type A personality. Mentioned researchers found that AMI patients presented a higher degree of behavior corresponding to Type A personality and also showed a higher degree of anxiety and stress in comparison to healthy controls (Miličić et al., 2016).

The presence of certain factors may encourage physicians to reach for alcohol and contribute to the occurrence of problematic habits related to its excessive consumption. Like no other profession, physicians are exposed to burnout and mental health problems, including depression and anxiety, which are among the reasons for seeking unfavorable ways of coping with them. Identifying excessive alcohol use in the described group is difficult, but considering the long-term effects of alcohol use on cognitive processes (including judgment, mood, impulse control and learning), as well as health effects (circulatory system diseases, cancer and liver cirrhosis), it is extremely important. The research of Crovetto M. et al. showed that medical students who consumed alcohol had a generally higher score in terms of an unhealthy diet compared to non-consumers (Crovetto et al., 2022). The meta-analysis by Wilson J. et al. showed that problematic alcohol use by physicians increased over the years from 16.3% in 2006–2010 to 26.8% in 2017–2020. As many as 7 out of 19 studies analyzed by the aforementioned authors showed that problematic alcohol use is more common in men than in women, which is confirmed by the results obtained in our own study (Wilson et al., 2022). The study by Oreskovich MR. et al. showed that the scale of problematic alcohol use or alcohol dependence, assessed on the basis of the AUDIT-C test results, significantly decreased with the years of professional practice, which was also confirmed by the results of our own study (Oreskovich et al., 2015).

A common problem in a doctor’s work is the lack of or significant limitation of the possibility of resting, eating and drinking during working hours. This condition is associated with stress for the body and the possibility of developing adverse complications. There is growing empirical evidence suggesting a relationship between the well-being of doctors and their ability to provide high-quality health care. Studies by Lemaire J et al. have shown that poor nutrition in the workplace has a significant negative impact on the personal well-being and professional performance of doctors, posing a threat to the quality of services and the organization of health care and the performance of the medical profession (Lemaire et al., 2011). Despite this, doctors are often unable to take care of their well-being in this regard.

All mentioned factors as well as high demat work in the healthcare sector can lead to burnout syndrome. Research by Angelopoulos K. et al. showed that among the students from their research approximately 33.5% showed a high risk or tendency to develop burnout syndrome, of which 11.75% were in the high-risk group and already suffered from this syndrome (Angelopoulos et al., 2025). Fatoke B. et al. showed that 14% of 3,400 junior physicians reported mild burnout, 18% moderate burnout, 29% high burnout, and 39% very high burnout. No significant differences in burnout were found between surgical and nonsurgical specialties (*F* = 0.951, *p* = 0.386). However, age, physical and mental health, social relationships, and environment were significant predictors of burnout (*p* < 0.01) (Fatoke et al., 2025). Selected individual characteristics are strong predictors of burnout. Angelini G. meta-analysis showed that higher levels of neuroticism and lower levels of agreeableness, significant conscientiousness, as well as extraversion and openness were associated with lower levels of self-esteem and are associated with a higher risk of developing burnout. Therefore, workers with those features should be monitored and cared in order to prevent future problems related do described syndrome (Angelini, 2023).

The limitations of the study include the unevenness of the groups compared and the small number of respondents for a survey. Moreover, self-reported data can be biased either consciously or unconsciously by respondents who provide information that can inaccurately reflect their true behaviors, attitudes, or experiences. This discrepancy can arise from social desirability to present oneself in a favorable light. Cross-sectional studies are difficult to conduct, taking into account possible recall bias or cohort effects. Another challenge is to take into account the influence of some factors used in the analysis, such as the age or gender of the participants, which could potentially and, regardless of their personality type, influence the unhealthy behaviors. However, it should be emphasized that conducting surveys involving physicians is a difficult task due to the waiting time for a response and the general reluctance of physicians to share, even anonymously, data about their own lives. The conducted study, therefore, becomes important and allows for concluding the discussed topic, and may also indicate the direction of further research and become the basis for developing educational and corrective actions related to the anti-health behaviors.

## 6 Conclusion

The occurrence of Type A personality was associated with, among others: worse eating habits, more frequent sleep deprivation and exposure to stress and low physical activity. There is a need to conduct educational activities, not only among doctors and other health services members, but most importantly at an early school stage regarding the benefits of following healthy lifestyle recommendations and, consequently, limiting the development of diseases and their complications.

## Data Availability

The raw data supporting the conclusions of this article will be made available by the authors, without undue reservation.
