# Supplementary material for: The phenomenon of Type A and B personality prevalence and their correlation to the anti-health behavior of Polish physicians
Source: Front Psychol. 2025 Aug 12;16:1608564. doi: 10.3389/fpsyg.2025.1608564 (PMC12379021; doi:10.3389/fpsyg.2025.1608564)
Supplement: Supplementary file 1 [file Table_1.docx]

Supplementary material for article entitled:

**The phenomenon of Type A and B personality prevalence and their correlation to the anti-health behaviour of Polish physicians**

**Tab. S1.** Characteristics of the study group including dietary assessment

| **Study group of physicians (832; 100%)** | | | | | | |
| --- | --- | --- | --- | --- | --- | --- |
| **Variables** | | | | | | **n (%)** |
| **Number of meals consumed per day** | | | | | 4-5 | 377 (45.31%) |
|  |  |  |  |  | 3 | 382 (45.91%) |
|  |  |  |  |  | <3 | 73 (8.77%) |
| **Occurrence of breaks between meals >5 hours** | | | | | Yes | 505 (60.70%) |
|  |  |  |  |  | No | 327 (39.30%) |
| **Presence of dairy products in meals [number of meals]** | | | | | 2-3 | 203 (24.40%) |
|  |  |  |  |  | 1 | 546 (65.63%) |
|  |  |  |  |  | 0 | 83 (9.98%) |
| **Presence of products providing animal protein other than dairy in meals [number of meals]** | | | | | 3-4 | 160 (19.23%) |
|  |  |  |  |  | 2 | 453 (54.45%) |
|  |  |  |  |  | 1 or 0 | 219 (26.32%) |
| **Presence of vegetables or fruits in meals [number of meals]** | | | | | 3-4 | 263 (31.61%) |
|  |  |  |  |  | 2 | 539 (64.78%) |
|  |  |  |  |  | 1 or 0 | 30 (3.61%) |
| **Presence of vegetables or fruits rich in vitamin C and carotenes in meals [number of meals]** | | | | | 3 | 115 (13.82%) |
|  |  |  |  |  | 2 | 652 (78.37%) |
|  |  |  |  |  | 1 | 65 (7.81%) |
| **Presence of salads in meals [number of meals]** | | | | | 2 | 89 (10.70%) |
|  |  |  |  |  | 1 | 668 (80.59%) |
|  |  |  |  |  | 0 | 75 (9.01%) |
| **Presence of dark bread or coarse groats in meals [number of meals]** | | | | | 2 | 215 (25.84%) |
|  |  |  |  |  | 1 | 468 (56.25%) |
|  |  |  |  |  | 0 | 149 (17.91%) |
| **The need for changes in the meals consumed** | | | | | Yes | 823 (100%) |
|  |  |  |  |  | No | 0 (0%) |
| **M** | **SD** | **Me** | **Q1** | **Q3** | **Min.** | **Max** |
| 16,75 | 2,68 | 17,00 | 15,00 | 19,00 | 4,00 | 25,00 |

**Explanation of abbreviations:** M – mean, SD – standard deviation, Me – median, Q1 – lower quartile, Q3 – upper quartile, Min. – minimum value, Max. – maximum value.

Almost 46% (382; 45.91%) of the examined group of doctors declared eating 4-5 meals a day, and the presence of vegetables or fruit in 2 meals – less than 65% (539; 64.78%). Unfortunately, in all respondents (823; 100%) the applied questionnaire for assessing the diet showed a necessity of diet changes.

**Tab. S2**. Characteristics of the study group, including the analysis of differences in the number of points obtained in *The Point Scale of the Diet Assessment* and personality type A/B

| **Study group of physicians (823; 100%)** | | | | | | | | |
| --- | --- | --- | --- | --- | --- | --- | --- | --- |
| **Variables** | | **Descriptive statistics** | | | | | **H** | **p** |
|  |  | **M** | **SD** | **Me** | **Q1** | **Q3** |  |  |
| **Personality type** | A | 16.45 | 2.74 | 16.00 | 15.00 | 18.00 | 8.900 | 0.01* |
|  | Intermediate | 17.03 | 2.44 | 17.00 | 15.00 | 19.00 |  |  |
|  | B | 16.90 | 2.73 | 17.00 | 15.00 | 19.00 |  |  |
| **Post-hoc:** A : B – p=0.02 | | | | | | | | |

**Explanation of abbreviations:** M – mean, SD – standard deviation, Me – median, Q1 – lower quartile, Q3 – upper quartile, H – Kruskal-Wallis test result, p – statistical significance, * - statistically significant result.

Analyses of differences showed that, taking into account the number of points in the diet assessment questionnaire, significantly lower results, and consequently a worse diet, were obtained by physicians with type A personality.

**Tab. S3.** Characteristics of the study group, including descriptive statistics of points obtained in individual scales of the *My Eating Habits Questionnaire* and the total number of obtained points

| **Variables** | ***Habitual overeating*** | ***Emotional overeating*** | ***Dietary restrictions*** | **Total points** |
| --- | --- | --- | --- | --- |
| **Mean** | 2,88 | 3,82 | 2,99 | 9,69 |
| **Standard deviation** | 2,42 | 2,45 | 2,29 | 5,75 |
| **Median** | 2,00 | 3,00 | 3,00 | 9,00 |
| **Upper quartile** | 4,00 | 5,00 | 5,00 | 14,00 |
| **Lower quartile** | 1,00 | 2,00 | 1,00 | 5,00 |
| **Dominant** | 2 | 2 | 1 | 4 |
| **Dominant number** | 177 | 140 | 142 | 67 |
| **Minimum value** | 0 | 0 | 0 | 0 |
| **Maximum value** | 10,00 | 10,00 | 10,00 | 29,00 |
| **p S-W** | <0,001 | <0,001 | <0,001 | <0,001 |

**Explanation of abbreviations:** p S-W – Shapiro-Wilks distribution normality test value

The average number of points obtained was as follows: *Habitual overeating* -2.88±2.42, *Emotional overeating* – 3.82±2.45, *Dietary restrictions* – 2.99±2.29, and the total number of points – 9.69±5.75.

The characteristics of the study group of physicians, taking into account the comparison of eating habits measured with *My Eating Habits Questionnaire* and socio-demographic characteristics, are presented in Table 6. Post-hoc analyses of the Kruskal-Wallis test for significant multiple comparisons are presented in Table 7 and 8.

**Tab. S4.** Characteristics of the studied group of physicians, taking into account eating habits and socio-demographic characteristics

| **Variables** | **Eating habits of the surveyed group of physicians (823; 100%)** | | | | | | | |
| --- | --- | --- | --- | --- | --- | --- | --- | --- |
|  | ***Habitual overeating***  **Me (Q1-Q3)** | p | ***Emotional overeating* Me (Q1-Q3)** | p | ***Dietary restrictions***  **Me (Q1-Q3)** | p | **Sum of points**  **Me (Q1-Q3)** | p |
| **Sex** | | | | | | | | |
| Women | 2 (1-4) | 0.740^A^ | 4 (2-6) | 0.002^A*^ | 3 (1-6) | 0,003^A*^ | 9 (6-14) | 0.016^A*^ |
| Men | 2 (1-4) |  | 3 (2-5) |  | 2 (1-4) |  | 8 (4-13) |  |
| **Age [years]** | | | | | | | | |
| ≤30 | 3 (1-5) | <0.001^B*^ | 4 (2-6) | 0.02^B*^ | 3 (1-5) | 0358 ^B^ | 9 (6-14) | 0.024^B*^ |
| 31-40 | 3 (2-4) |  | 3 (2-6) |  | 2 (1-4) |  | 9 (5-14) |  |
| 41-50 | 2 (1-3) |  | 3 (1-5) |  | 3 (1-5) |  | 8 (4-12) |  |
| ≥51 | 2 (1-3) |  | 4 (2-6) |  | 3 (1-5) |  | 9 (5-14) |  |
| **Length of service [years]** | | | | | | | | |
| 1-10 | 3 (1-5) | <0.001^B*^ | 4 (2-6) | 0.422^B^ | 2 (1-4) | 0.242^B^ | 9 (5-14) | 0.187^B^ |
| 11-20 | 2 (1-4) |  | 3 (2-6) |  | 2 (1-5) |  | 8 (4-13) |  |
| 21-30 | 2 (1-4) |  | 4 (2-5) |  | 3 (1-5) |  | 9 (5-14) |  |
| >31 | 2 (1-4) |  | 4 (2-5) |  | 3 (1-4) |  | 9 (6-12) |  |
| **Ward type** | | | | | | | | |
| Surgical | 2 (1-5) | 0.160^A^ | 3 (2-6) | 0.820^A^ | 2 (1-4) | 0.03^A*^ | 9 (5-14) | 0.800^A^ |
| Non-surgical | 2 (1-4) |  | 3 (2-5) |  | 3 (1-5) |  | 9 (5-13) |  |
| **Additional employment** | | | | | | | | |
| Yes | 2 (1-4) | 0.237^A^ | 4 (2-6) | 0.105^A^ | 3 (1-5) | 0.934^A^ | 9 (5-13) | 0.326^A^ |
| No | 2 (1-4) |  | 3 (2-5) |  | 2 (1-5) |  | 8 (5-14) |  |
| **Working on duty** | | | | | | | | |
| Yes | 2 (1-4) | 0.023^A^ | 3 (2-5) | 0.802^A^ | 3 (1-4) | 0.292^A^ | 9 (5-14) | 0.620^A^ |
| No | 2 (1-3) |  | 4 (2-6) |  | 3 (1-6) |  | 8 (5-13) |  |

**Explanation of abbreviations:** Me – median, Q1-Q3 – interquartile range, p – statistical significance, A – statistical significance from the Mann-Whitney U test, B-statistical significance from the Kruskal-Wallis test, * - statistically significant result.

**Tab. S5.** Results of post-hoc analyses between age groups and points obtained in *Habitual* and *Emotional overeating* scales as well as total sum of points

| ***Habitual overeating*** | **Age** | | **≤30** | | **31-40** | | **41-50** | | **≥51** | | |
| --- | --- | --- | --- | --- | --- | --- | --- | --- | --- | --- | --- |
|  | **≤30** | | - | | NS | | <0,001 | | 0,002 | | |
|  | **31-40** | | NS | | - | | <0,001 | | <0,001 | | |
|  | **41-50** | | <0,001 | | <0,001 | | - | | NS | | |
|  | **≥51** | | 0,002 | | <0,001 | | NS | | - | | |
| ***Emotional overeating*** | **Age** | | **≤30** | | **31-40** | | **41-50** | | **≥51** | |  |
|  | **≤30** | | - | | NS | | 0,03 | | NS | |  |
|  | **31-40** | | NS | | - | | N | | NS | |  |
|  | **41-50** | | 0,03 | | NS | | - | | NS | |  |
|  | **≥51** | | NS | | NS | | NS | | - | |  |
| ***Sum of points*** | | **Age** | | **≤30** | | **31-40** | | **41-50** | | **≥51** | |
|  |  | **≤30** | | - | | NS | | 0,02 | | NS | |
|  |  | **31-40** | | NS | | - | | NS | | NS | |
|  |  | **41-50** | | 0,02 | | NS | | - | | NS | |
|  |  | **≥51** | | NS | | NS | | NS | | - | |

**Explanation of abbreviations:** NS – no statistical significance.

**Tab. S6.** Results of post-hoc analyzes between categorized years of service and points obtained in *Habitual overeating* subscale

| ***Habitual overeating*** | **Length of service** | **1-10** | **11-20** | **21-30** | **≥31** |
| --- | --- | --- | --- | --- | --- |
|  | **1-10** | - | <0,001 | 0,003 | 0,04 |
|  | **11-20** | <0,001 | - | NS | NS |
|  | **21-30** | 0,003 | NS | - | NS |
|  | **≥31** | 0,04 | NS | NS | - |

**Explanation of abbreviations:** NS – no statistical significance.

The Mann-Whitney U test showed that women had a greater tendency to emotional overeating (Z=-3.076; p=0.002) and dietary restrictions (Z=-3.02; p=0.003) than men. In addition, the aforementioned test showed that doctors working in non-surgical wards had a greater tendency to dietary restrictions (Z=2.1; p=0.03), and working on duty was associated with habitual overeating (Z=-2.28; p=0.023). The Kruskal-Wallis test showed that people aged ≤30 years had a greater tendency to habitual overeating than respondents aged 41-50 (p<0.001) and ≥51 years (p=0.002), and also revealed that people who had been working for 1 to 10 years had a greater tendency to habitual overeating than the remaining respondents (11-20 – p<0.001, 21-30 – p=0.003, ≥31 – p=0.04).
